# Gaps in the evidence for prevention and treatment of maternal anaemia: a review of systematic reviews

**DOI:** 10.1186/1471-2393-12-56

**Published:** 2012-06-24

**Authors:** Jacqui A Parker, Filipa Barroso, Simon J Stanworth, Helen Spiby, Sally Hopewell, Carolyn J Doree, Mary J Renfrew, Shubha Allard

**Affiliations:** 1 Department of Obstetric Anaesthesia, John Radcliffe Hospital, Headley Way, Headington, Oxford OX3 9BQ, UK; 2 Department of Haematology, Barts and London Hospitals NHS Trust & NHS Blood & Transplant, London, UK; 3 Department of Haematology, John Radcliffe Hospital, NHS Blood & Transplant/Oxford Radcliffe Hospitals Trust, University of Oxford, Oxford, UK; 4 School of Nursing, Midwifery and Physiotherapy, University of Nottingham, Nottingham, UK; 5 Systematic Review Initiative, NHS Blood & Transplant, John Radcliffe Hospital, Oxford, UK; 6 Mother and Infant Research Unit, Dept of Health Sciences, University of York, York, UK

## Abstract

**Background:**

Anaemia, in particular due to iron deficiency, is common in pregnancy with associated negative outcomes for mother and infant. However, there is evidence of significant variation in management. The objectives of this review of systematic reviews were to analyse and summarise the evidence base, identify gaps in the evidence and develop a research agenda for this important component of maternity care.

**Methods:**

Multiple databases were searched, including MEDLINE, EMBASE and *The Cochrane Library.* All systematic reviews relating to interventions to prevent and treat anaemia in the antenatal and postnatal period were eligible. Two reviewers independently assessed data inclusion, extraction and quality of methodology.

**Results:**

27 reviews were included, all reporting on the prevention and treatment of anaemia in the antenatal (n = 24) and postnatal periods (n = 3). Using AMSTAR as the assessment tool for methodological quality, only 12 of the 27 were rated as high quality reviews. The greatest number of reviews covered antenatal nutritional supplementation for the prevention of anaemia (n = 19). Iron supplementation was the most extensively researched, but with ongoing uncertainty about optimal dose and regimen. Few identified reviews addressed anaemia management post-partum or correlations between laboratory and clinical outcomes, and no reviews reported on clinical symptoms of anaemia.

**Conclusions:**

The review highlights evidence gaps including the management of anaemia in the postnatal period, screening for anaemia, and optimal interventions for treatment. Research priorities include developing standardised approaches to reporting of laboratory outcomes, and information on clinical outcomes relevant to the experiences of pregnant women.

## Background

Anaemia is the most common disorder of pregnancy with estimates of global prevalence reaching over 40% [1]. Iron deficiency is the commonest cause of anaemia but other causes include nutritional deficiencies, bone marrow suppression and haemolytic or hereditary diseases. Blood loss during, as well as shortly after, birth may further contribute to postpartum anaemia.

The consequences of anaemia in pregnancy are potentially far-reaching, both for mother and infant. Several studies have identified anaemia as a risk factor for intrauterine fetal death, premature birth, low birth weight and other adverse neonatal outcomes [2,3]. Some suggest a link between maternal anaemia in pregnancy and later developmental problems in children [4,5]. Postpartum maternal anaemia may be associated with breathlessness, lethargy, infection, lactation failure and depression [2,5-7]. Despite this, there is evidence of significant variation in practice and sub-optimal management of maternal anaemia [8-10], possibly due to a lack of consistent, detailed information derived from good quality evidence.

We carried out a review of systematic reviews to summarise the evidence base, to identify gaps in the evidence and to develop a research agenda for this important component of maternity care. The primary focus of this review was the range of interventions used to prevent and treat anaemia in pregnancy and up to 1 year postpartum. The project focussed on systematic reviews of randomised controlled trials (RCTs) as the most robust form of study design to address the effectiveness of interventions. Given the expected clinical diversity in practices and populations, we choose not to include reviews that focussed on resource poor countries. This review of systematic reviews followed principles set out in the Cochrane Handbook [11].

## Methods

### Searching

A comprehensive search was performed in December 2009, and updated on 11 May 2011, using the bibliographic databases: MEDLINE (1950 onwards), EMBASE (1980 onwards), *The Cochrane Library* Issue 5, 2011, CINAHL (1982 onwards), British Nursing Index (1994 onwards), MIDIRS: Maternity and Infant Care, UKBTS SRI Transfusion Evidence Library, BMJ Clinical Evidence and Open SIGLE (System for Grey Literature in Europe). The review authors also checked the reference lists of all identified trials, relevant review articles and current treatment guidelines for further literature. Details of the search strategies are provided in Additional file 1. Searches were not restricted by language or publication date.

### Inclusion/Exclusion criteria

All systematic reviews of interventions to prevent and treat anaemia in women, of any ethnicity or age group, during the maternity period (conception to 1 year postpartum), were eligible for inclusion. Systematic reviews from resource poor countries that were not considered relevant to health care systems in developed countries, and reviews focussing exclusively on women with inherited disorders of haemoglobin synthesis, e.g. thalassaemia and sickle-cell disease, were excluded as these were not the intended focus of our review. Systematic reviews focussing on the intrapartum period (onset of labour to completion of third stage) were also excluded as such reviews are primarily concerned with specialised obstetric interventions to reduce blood loss rather than the management of anaemia per se.

All interventions and outcomes relevant to prevention and treatment of maternal anaemia in pregnancy and the postpartum, were considered. Outcome measures did not form part of the eligibility criteria for inclusion. To be included as a systematic review, the study had to use a systematic search attempting to identify all studies meeting their eligibility criteria.

### Data collection and analysis

One reviewer screened all titles/abstracts for relevance and excluded citations were checked by a second reviewer. Only irrelevant titles/abstracts were excluded at this stage (e.g. techniques for pelvic surgery in subfertility). The remaining studies were assessed for inclusion on the basis of their full text, using the criteria indicated above. Data inclusion, extraction and quality of methodology were independently assessed by two reviewers and any disagreements resolved by consensus with a third experienced reviewer with subject specific expertise.

Included studies were initially mapped onto a table (Table 1) demonstrating the area of anaemia management (i.e. prevention or treatment) and divided into the following categories - *antenatal*: from conception to the onset of labour; *postnatal*: from completion of the third stage to 1 year postpartum. This table was used to identify areas that have been extensively researched and those with lack of data (evidence gaps). Analysis included a narrative synthesis, presented in tabulated and text form, of all included studies. Conclusions were based on patterns of results across reviews.

**Table 1:** Mapping of included systematic reviews

	**Interventions around prevention and screening**		**Interventions around treatment**	
**Antenatal**	**Nutrition supplementation**	**Total** = **19**	**Nutrition supplementation**	**Total** = **3**
	Multiple/ micronutrients	Fishman, S (2000)*	Iron &/or folate	*Mathews, F (1996)*
		*Villar, J (2003)*		Rasmussen, K (2001)*
		*Haider, B (2006) ↑*		Reveiz, L (2007)* ↑
		*Haider, B (2011) ↑*		
	Vitamin A	*Faisel, H (2000)*		
		*Van den Broek, N (2002) ↑*		
	Vitamin E	*Rumbold, A (2005) ↑*		
	Vitamin C	*Rumbold, A (2005) ↑*		
	Iron & folic acid only	*Gulmezoglu, M (1997)*		
		*Kulier, R (1998)*		
		*De Onis, M (1998)*		
		*Milman, N (1999)*		
		Rasmussen, K (2001)*		
		*Sloan, N (2002)*		
		AHRQ (2006)*		
		Reveiz, L (2007)* ↑		
		*Peña-Rosas, J (2009) ↑*		
		*Macedo, A (2010)*		
		*Yakoob, M (2011) ↑*		
	**Organisation of antenatal care**			
		**Total** = **5**		
		*Scholl, T (1994)*		
		*Carroli, G &Villar, J (2001) ↑*		
		*Villar, J (2001) ↑*		
		*Carroli, G &Rooney, C (2001)*		
		*Dodd, J (2007) ↑*		
**Postnatal (up to 1 year)**	**Iron**	**Total** = **1**	**Iron &/or erythropoietin**	**Total** = **3**
		Fishman, S (2000)*		*Dodd, J (2004) ↑*
				*Kotto-Kome, A (2004)*
				AHRQ (2006)*

* Systematic review included in more than one row/column.

↑ Systematic review categorised as high quality.

### Assessment of methodological quality

Appraisal of the quality of the systematic reviews was determined using AMSTAR; a measurement tool to assess the methodological quality of systematic reviews [12] (Appendix 2, see Additional file 2). The tool consists of 11 items, in the form of questions, about the methodological quality of each systematic review. The 11 items were assessed for each review and the total number of positive answers for each was documented. The reviews were then divided into the following categories - *high quality*: 9 or more positive answers; *intermediate quality*: 5–8 positive answers; *low quality*: 4 or less positive answers. This provided an overall rating of each systematic review that could be used in the interpretation of the narrative results.

## Results

### Search results

**Figure 1:**
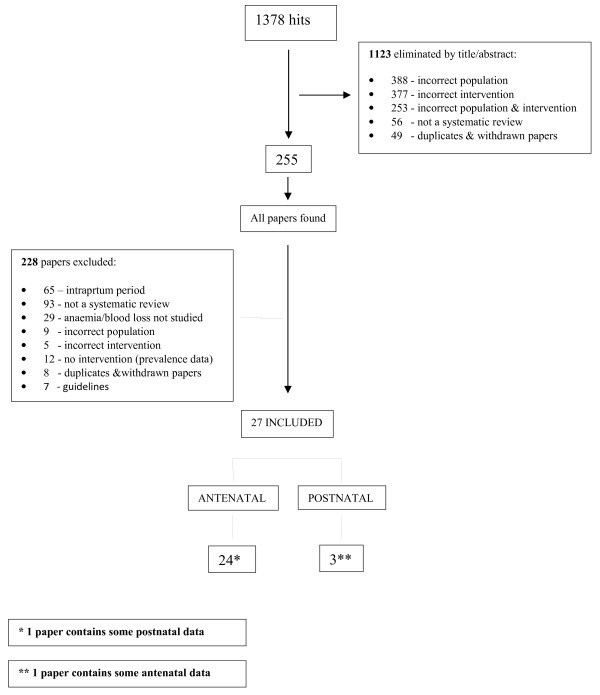
**Flow diagram.** A diagram mapping out the number of records identified, included and excluded, during the review process and the reasons for exclusions.

The search identified 1,378 records of which the majority (n = 1,123) were eliminated by title/abstract (see Figure 1). 255 records were reviewed as full text articles, with 163 excluded because they were not a systematic review or did not fulfil the inclusion criteria. A further 65 were excluded as they focussed on the intrapartum period.

The remaining 27 reviews formed the basis of the analysis (Appendix 3 shows complete list of references, see Additional file 3). Reviews were first mapped according to the time period (antenatal or postnatal) and area of anaemia management (prevention or treatment) that was the predominant focus of each review (Table 1). The total numbers in each section differ slightly from those in Figure 1 as some systematic reviews mapped to more than one section.

### Prevention and treatment of anaemia in the antenatal and postnatal period

All identified systematic reviews reported on the prevention and treatment of anaemia in the antenatal (n = 24) and postnatal period (n = 3). There was some crossover between the antenatal and postnatal period in two of the systematic reviews. All were published between 1994 and 2011, with the majority (n = 21) being published from 2000 to 2011 (see Additional file 4: Table S2). Only three reviews exclusively included RCTs from the developed world [13-15]. The largest number covered antenatal nutritional supplementation for the prevention of anaemia (n = 19), including the use of iron and folic acid. Five reviews addressed the role of antenatal care organisation in preventing anaemia. Only a small number of reviews reported on interventions for the treatment of antenatal anaemia (n = 3) and for the prevention (n = 1) and treatment (n = 3) of postnatal anaemia.

### Assessment of methodological quality

Three of the 27 systematic reviews reported data for all AMSTAR items (Table 2). The most common items that no data was reported for were item 5 (providing a list of studies, both included and excluded) and item 10 (the assessment of publication bias). Unavailable data was reported as CA, and nine reviews had this response at least once. As a result of the AMSTAR assessment, 12 systematic reviews were categorised as high quality, four as intermediate quality and 11 as low quality systematic reviews. Of the 12 high quality reviews, 11 focussed predominantly on antenatal prevention of anaemia (8 on nutritional supplementation, 3 on organisation of antenatal care). Only one high quality review focussed predominantly on the postnatal period, and this was for the treatment of anaemia rather than its prevention.

**Table 2:** Appraisal of methodological quality

**Paper**	**AMSTAR Question**											**No. of Y**	**Overall Quality**
	**1**	**2**	**3**	**4**	**5**	**6**	**7**	**8**	**9**	**10**	**11**		
Fishman, S (2000) [16]	Y	N	N	N	N	Y	N	N	NA	N	N	**2**	**Low**
Villar, J (2003) [17]	Y	CA	Y	CA	Y	Y	Y	N	Y	N	N	**6**	**Intermediate**
Haider, B (2006) [18]	Y	Y	Y	Y	Y	Y	Y	Y	Y	Y	Y	**11**	**High ↑**
Haider, B (2011) [19]	Y	Y	Y	Y	Y	Y	Y	Y	Y	N	Y	**10**	**High ↑**
Faisel, H (2000) [20]	Y	N	Y	Y	N	N	N	N	NA	N	N	**3**	**Low**
Van den Broek, N (2002) [21]	Y	Y	Y	Y	Y	Y	Y	Y	Y	N	Y	**10**	**High ↑**
Rumbold, 2005 (Vit E) [22]	Y	Y	Y	Y	Y	Y	Y	Y	Y	N	Y	**10**	**High ↑**
Rumbold, 2005 (Vit C) [23]	Y	Y	Y	Y	Y	Y	Y	Y	Y	N	Y	**10**	**High ↑**
Gulmezoglu, M (1997) [24]	Y	CA	Y	CA	N	Y	Y	Y	NA	N	Y	**6**	**Intermediate**
Kulier, R (1998) [25]	Y	N	Y	CA	N	Y	Y	Y	Y	N	N	**6**	**Intermediate**
De Onis, M (1998) [26]	CA	CA	N	CA	N	Y	Y	Y	Y	N	N	**4**	**Low**
Milman, N (1999) [27]	N	N	N	N	N	Y	N	N	NA	N	N	**1**	**Low**
Rasmussen, K (2001) [28]	Y	N	N	N	N	Y	Y	Y	NA	N	N	**4**	**Low**
Sloan, N (2002) [29]	Y	CA	N	N	N	N	N	N	Y	N	Y	**3**	**Low**
AHRQ, 2006 [30]	Y	N	Y	CA	N	N	Y	Y	NA	N	N	**4**	**Low**
Reveiz, L (2007) [31]	Y	Y	Y	Y	Y	Y	Y	Y	Y	N	Y	**10**	**High ↑**
Pena-Rosas, J (2009) [32]	Y	Y	Y	Y	Y	Y	Y	Y	Y	Y	Y	**11**	**High ↑**
Macedo, A (2010) [33]	Y	CA	Y	Y	N	Y	Y	Y	NA	N	Y	**7**	**Intermediate**
Yakoob, M (2011) [34]	Y	Y	Y	Y	Y	Y	Y	Y	Y	N	Y	**10**	**High ↑**
Scholl, T (1994) [13]	CA	N	N	N	N	Y	N	N	CA	N	Y	**2**	**Low**
Carroli G &Villar J ( 2001) [35]	Y	Y	Y	Y	N	Y	Y	Y	Y	Y	Y	**10**	**High ↑**
Villar, J (2001) [36]	Y	Y	Y	Y	Y	Y	Y	Y	Y	Y	Y	**11**	**High ↑**
Carroli G &Rooney C (2001) [37]	Y	N	Y	Y	N	N	N	N	NA	N	N	**3**	**Low**
Dodd, J (2007) [38]	Y	Y	Y	Y	Y*	Y*	Y*	Y*	Y*	CA	Y	**10**	**High ↑**
Mathews, F (1996) [39]	Y	N	Y	N	N	N	Y	Y	NA	N	N	**4**	**Low**
Dodd, J (2004) [14]	Y	Y	Y	Y	Y	Y	Y	Y	Y	N	Y	**10**	**High ↑**
Kotto-Kome, A (2004) [15]	Y	N	N	N	N	Y	N	N	Y	N	N	**3**	**Low**

* Review did not include any studies therefore decision based on methodology.

*Y* = Yes.

*N* = No.

*NA* = Not applicable.

*CA* = Cannot answer.

Quality categories:.

Low = 4 or less Y answers.

Intermediate = 5–8 Y answers.

High ↑ = 9 or more Y answers.

Through the text we have indicated the quality of the review alongside its findings/recommendations in order to allow the reader to consider the findings/recommendation in light of the review’s measured quality.

### The antenatal period: prevention of anaemia

#### Use of iron and/or folate (n = 12 reviews)

##### Laboratory outcomes

Twelve systematic reviews addressed the role of iron and/or folate in the prevention of anaemia [17,24-34]. These reviews included RCTs with diverse settings and included women known to be anaemic or iron deficient as well as non-anaemic women. All reviews concluded that iron supplementation increased haematological markers (haemoglobin or haematocrit), improved serum ferritin levels, and decreased the incidence of anaemia. Three reviews [27,32,33] (one high, one intermediate and one low quality) showed that such positive effects extended into the postnatal period, with improved maternal haemoglobin concentrations one month to eight weeks postpartum.

One systematic review of high quality [32] and one of low quality, [27] reported infant outcomes. In both, infants born to iron supplemented mothers had a higher serum ferritin at birth compared to non-supplemented women. Pena-Rosas 2009 [32] found the effect to be maintained for up to six months (MD 0.09 95% CI 0.05–0.13).

No systematic reviews provided definitive evidence on the optimum dose or regimen of iron administration. Studies assessed different doses of ferrous iron (daily dose 27–200 mg), duration of therapy and gestational age at which treatment commenced. Two high quality reviews [32,34] reported that the risk of anaemia at term was similar for supplementation with daily or intermittent iron alone, compared with iron and folic acid. One review, of low quality, Sloan 2002 [29] showed a positive dose response between iron supplementation and maternal haemoglobin concentrations, however no conclusion could be drawn in regard to optimal duration of therapy as higher doses of iron were used in studies with shorter therapy periods, thus confounding the results. Villar 2003 [17] reported on daily versus weekly or twice weekly supplementation with both iron and folic acid. This review included one RCT which demonstrated that daily supplementation resulted in significantly higher increases in haemoglobin, two trials which showed no difference and a meta-analysis of four trials which concluded that daily supplements of iron and folic acid were superior to weekly. However this meta-analysis included trials with significant heterogeneity and the review was only assessed to be of intermediate quality.

#### Clinical outcomes including adverse effects

We identified little information relating to clinical outcomes. Pena-Rosas 2009 [32] (a high quality review) found no significant difference in small for gestational age, birthweight, perinatal death, or admission to special care unit, among infants whose mothers received iron and folic acid supplementation or not. Data from three RCTs suggested that women receiving iron supplementation had a lower risk of receiving blood transfusion (RR 0.61; 95% CI 0.38–0.96). Macedo 2010 [33] (an intermediate quality review) identified two RCTs that showed improved birthweight in iron supplemented women, and one RCT that showed a reduction in preterm labour and perinatal mortality. Nevertheless heterogeneity between RCTs led the authors to conclude that no definitive link between iron supplementation and improvement in pregnancy outcomes could be made. One low quality systematic review [28] reported that the supplementation of non-anaemic women with iron and/or folic acid did not affect birth weight or duration of pregnancy.

Few systematic reviews reported on adverse events associated with the prevention of anaemia. Three reviews [29,32,33] (one high, one intermediate and one low quality) concluded that adverse events, primarily gastrointestinal (nausea and epigastric pain), were more common with iron than placebo, and with higher versus lower doses of iron supplementation.

### Use of different supplements (n = 7 reviews)

#### Laboratory outcomes

Seven systematic reviews (four of high quality) investigated the effects of micronutrients other than iron and folic acid [16,18-23] and found no evidence to support the use of vitamin B12, vitamin C, calcium or zinc to prevent anaemia during pregnancy. Vitamin C is reported to enhance absorption of non-haem iron and mobilisation of iron, but the reviews highlighted uncertainty in the optimal role and schedule for Vitamin C supplementation on outcomes such as maternal anaemia or other measures of iron status [23].

Four of the seven systematic reviews assessed the role of vitamin A [16,17,20,21], although only one was of high quality. [21] All included a single RCT (Suharno 1993 [40]) which demonstrated higher mean haemoglobin concentrations and lower prevalence of anaemia at birth for women who had received a combination of vitamin A and iron, compared to women who received vitamin A or iron alone. However, these findings were not supported by the other included RCTs. The dose and duration of vitamin A supplementation varied from 2,400–60,000 μg/day, and between 6–28 weeks. It was not clear at what gestational age supplementation was started. In one low quality review [16] riboflavin was reported to enhance the haematological response to iron supplementation (5–9 mg of riboflavin for 6 weeks). The role of multivitamins compared to iron and folic acid supplementation was less clear [16,18,19].

#### Clinical outcomes

Haider 2011 [19] (a high quality review) concluded that the use of multivitamins significantly reduced the incidence of small for gestational age infants when compared to iron and folate supplementation alone (RR = 0.91 95% CI 0.86–0.96). No other review reported any clinical outcomes for mother or infant.

### Organisation of antenatal care (n = 5 reviews)

Five systematic reviews focussed on the organisation of antenatal services [13,35-38], and three of the five were of high quality. One, Dodd 2007 [38] (a high quality review) compared maternal and infant outcomes for women with multiple pregnancies managed in dedicated versus routine antenatal clinics but did not identify any relevant RCTs. Three systematic reviews [35-37] (two of which are high quality) addressed the number and frequency of antenatal care visits; all included a single RCT that incorporated maternal anaemia as an outcome. There was no difference in risk of maternal anaemia at term between standard and reduced visit antenatal care. A fifth, Scholl 1994 [13] (a low quality review) identified two RCTs that found no difference in risk of anaemia between dedicated antenatal care programmes for adolescents (including social support, schooling support, more intensive health promotion and education) and standard antenatal care.

### The antenatal period: treatment of anaemia

#### Use of iron (n = 3 reviews)

##### Laboratory outcomes

We identified one systematic review [31] (high quality) which included information on the use of iron and laboratory outcomes in the treatment of anaemia in the antenatal period. Overall treatment with iron, regardless of dose, method of administration, or frequency, consistently increased levels of maternal haemoglobin, haematocrit and serum ferritin, measured at different times during pregnancy. In general, daily iron supplementation was found to be more effective than weekly or twice weekly regimes, at increasing the mean haemoglobin. Parenteral iron preparations (intramuscular and intravenous) when compared to oral iron, were reported to improve haematological markers and reduce the incidence of anaemia at term. No difference was found in the number of women who attained haemoglobin greater than 11 g/dl, when comparing the combination of intravenous iron sucrose with recombinant human erythropoietin.

#### Clinical outcomes

We identified three systematic reviews examining the use of iron and clinical outcomes in the treatment of anaemia in the antenatal period. Reveiz 2007 [31], the only high quality systematic review in this area, found no evidence that increases in haematological markers led to any clinical improvements for mother or infant. However, very few of the RCTs assessed clinical outcomes and those that did were not sufficiently powered to detect any clinical effect. Rasmussen 2001 [28] (low quality) assessed the effect of antenatal iron and folic acid supplementation of anaemic women on birth weight and gestational age of infants and reported no statistically significant difference. The gestational age at which treatment commenced varied and was often not specified. Iron dosage and preparation also varied and ranged from 12–100 mg, which includes those doses that might be considered too low for clinical effect. The review by Mathews 1996 [39] (low quality) only included one RCT that evaluated routine iron supplementation versus targeted supplementation and found that mean gestational age and infant length at birth were greater in the routine supplement group but not statistically significant. There was no difference in mean birth weight or placental weight. No details of the dose regime were provided.

The incidence of gastrointestinal side effects was more common with oral iron (n = 244 RR 0.33 95% CI 0.15–0.74) [31]. Trials that compared daily with weekly or twice weekly regimes did not identify any benefit with regards to side effects other than constipation. Severe adverse events were more common with parenteral iron therapies [31]. Use of intravenous iron dextran was associated with higher risk of severe allergic reactions, but the authors were unable to establish the level of risk. Comparative studies of different formulations of intravenous iron were generally lacking, precluding more accurate assessments of risk of adverse reactions. A possible increased risk of venous thrombosis was noted in one review; one RCT suggested that this risk could be reduced by adding hydrocortisone (n = 30 RR 0.09 95% CI 0.01–1.51) [31].

### Use of different supplements

No reviews relating to laboratory or clinical outcomes with the use of micronutrients other than iron, in the treatment of anaemia during pregnancy, were identified.

### The postnatal period: prevention of anaemia (n = 1)

#### Laboratory outcomes

We identified one systematic review [16] (low quality) that looked at the effects of nutritional interventions to prevent anaemia in the postpartum. In a socially deprived population, multivitamins combined with folic acid administered from the second trimester to birth significantly increased the mean haemoglobin and haematocrit at six weeks postpartum, compared to multivitamins alone. In one trial, addition of 5 mg riboflavin to iron supplementation administered for six weeks postpartum augmented plasma levels of iron and ferritin when compared to iron alone.

### Clinical outcomes

No reviews reported clinical outcomes for the use of iron or micronutrients in the prevention of postpartum anaemia.

### The postnatal period: treatment of anaemia (n = 3)

#### Laboratory outcomes

Two systematic reviews (one high quality [14] and one low quality [15]) addressed the role of erythropoietin in combination with iron supplementation in tackling maternal anaemia in the first six weeks postpartum. They included nine RCTs, conducted in developed countries. Doses of erythropoietin ranged from 150–300 units/kg and frequency of administration varied from a single dose to a daily course over 15 days. Iron dosage ranged from 160–400 mg per day, with two trials randomising women to either intravenous or oral iron. The reviewers concluded that the combination of erythropoietin and iron raised haemoglobin more quickly than oral iron alone, but that by day 14 of administration the difference had disappeared.

### Clinical outcomes

Three systematic reviews addressed clinical outcomes in the treatment of maternal anaemia postpartum [14,15,30], yet only one was of high quality [14]. Two reviews [14,15] showed that women receiving erythropoietin were more likely to be lactating on discharge (two RCTs), with a decrease in postnatal depression scores in the erythropoietin treatment arm. No adverse events were recorded. The erythropoietin group demonstrated a significant increase in platelet count, but no increased thrombotic events. The AHRQ review [30] included a trial of iron supplementation versus placebo in mothers with iron deficiency anaemia. Infants of anaemic mothers had delays in their development, particularly in hand-to-eye coordination, and mothers were less engaged and responsive towards their babies. Supplementation was started at six to eight weeks postpartum, but the dose and duration of supplementation was unclear.

## Discussion

The main objective of this review was to identify gaps in the evidence for the prevention and treatment of maternal anaemia in pregnancy and up to 1 year postpartum, and to inform the research agenda. This topic remains a concern to health care professionals and public health specialists, as well as to women themselves, but the literature addressing this field is widely dispersed over a number of specialist publications/fields and has not been previously systematically collated.

One of the first striking gaps in the evidence base is in the management of maternal anaemia up to one year postpartum (Table 1). Secondly, across all identified systematic reviews, 25 included interventions for the prevention of anaemia, compared with only six reviews that included interventions for the treatment of anaemia. Thirdly, none of the reviews reported on the best methods of screening for anaemia. Finally, the majority of antenatal reviews evaluated the role of nutritional supplementation, including the use of iron which was consistently shown to improve haematological parameters and decrease the risk of anaemia at term. It was, however, impossible to make definitive statements about optimum dose or regimen, due to the heterogeneity between trials.

There was limited evidence to demonstrate that riboflavin, folic acid, vitamin A and the use of erythropoietin could enhance a response to iron. It is, therefore, unclear how or whether these other supplements should be used in current practice. No reviews examined the use of dietary modification or food support for the management of anaemia, and no reviews reported on cost-effectiveness of different approaches.

No reviews reported on clinical symptoms of anaemia and few reported on the correlation between laboratory and clinical outcomes. Despite evidence of improvement in haematological parameters with use of iron there was limited evidence to indicate that the reduction in antenatal maternal anaemia had any clinical impact for mother or infant, other than reducing the need for blood transfusion. There was some evidence to suggest that prompt treatment of postnatal anaemia may be beneficial in preventing postnatal depression and improving lactation, infant development and maternal bonding but the quality of this review was poor therefore results should be interpreted with caution. Data on adverse effects such as maternal exhaustion were lacking from the systematic review literature. A number of reviews reported on the use of different antenatal care programs for the prevention of antenatal anaemia, but there was no evidence that any type of program, including frequency of visits, was superior.

Recommendations do exist for best practice regarding the investigation and management of anaemia but these guidelines also seem to reflect many of the evidence uncertainties highlighted in this overview. The National Institute for Clinical Excellence (NICE) provides guidance on the management of antenatal care [41] and postnatal care [42], but in the postnatal guidelines there is no mention of the word ‘anaemia’. The RCOG green-top guideline for blood transfusion in obstetrics [43] highlights the importance of screening and treating anaemia, with oral or parental iron, in order to reduce the chances of later blood transfusion. Yet within these guidelines it is indicated that these are only ‘good practice points’ indicating that high-quality, systematically derived evidence is lacking.

Methodological quality of the included systematic reviews was variable with less than half assessed as being of high quality, thus consequently many of these systematic reviews are of limited utility for those seeking an evidence base to practice. 11 of the 12 high quality reviews fell across prevention of anaemia in the antenatal period and thus the findings and recommendations for practice from these reviews would be deemed to be more robust. Only one review of high quality was found for the postnatal period. Further systematic reviews in the postnatal period should meet the criteria for quality appraisal (PRISMA [44]) and provide information both on existing systematic reviews and the novel contribution of the new review.

A limitation of this review is the variation in practices and populations within and between countries, which may impact on the generaliseability of findings, although we partially addressed this by excluding reviews focussing on resource poor countries. A limitation of the methods used for this review of reviews is that, inevitably, there may be some recently published trials that have not yet been included in systematic reviews and thus are not included in our review. There is also some overlap in analyses of the same primary RCTs between systematic reviews; we have clearly identified where this has occurred.

The findings from this review of reviews have implications for new primary research and the research agenda. Anaemia management remains an uncertain area of research focus, despite its clinical importance. The identification or management of maternal anaemia was the main stated aim in only seven reviews [14-16,27,28,33,34] and overall, the number of RCTs that specifically reported on outcomes relevant to maternal anaemia was small (median 7, interquartile range 1–23).

A further priority is to develop standardised approaches to the reporting of laboratory outcomes as the heterogeneity of populations and settings, drug dosages and regimens and definitions of anaemia precluded quantitative analysis for many reviews. Outcomes relevant to the experiences and wellbeing of pregnant women and new mothers need to be included in future randomised controlled trials. Additional work is needed to identify the most effective way of managing anaemia in the postnatal period; validating an effective screening policy for both the ante and postnatal periods; and developing consensus on best practice for the treatment of maternal anaemia, including the dose, route and regimens of drugs including specifically iron. The role of universal supplementation strategies for iron has been evaluated in low-income postpartum women and needs wider consultation, taking into consideration all risks, including differences in rates of unintentional iron ingestion by young children in populations practicing selective versus universal iron supplementation to infants.

## Conclusions

Maternal anaemia is a common and clinically important problem however the results of this review indicate many gaps in the evidence. Such evidence gaps include the management of anaemia in the postnatal period, screening for anaemia, and optimal interventions for treatment. Research priorities include developing standardised approaches to reporting of laboratory outcomes, and information on clinical outcomes relevant to the experiences of pregnant women.

## Competing interests

No competing interests have been declared by the authors.

## Authors’ contributions

The review was conceived and planned by SS, HS and MJR; search strategy undertaken by CD; data extraction, analysis and methodological assessment completed by JP, FB and SH. All authors contributed to the final manuscript. All authors read and approved the final manuscript.

## Pre-publication history

The pre-publication history for this paper can be accessed here:

http://www.biomedcentral.com/1471-2393/12/56/prepub

## Supplementary Material

Additional file 1: Appendix 1 Search narrative and strategies. Click here for file

Additional file 2: Appendix 2 AMSTAR questions. Click here for file

Additional file 3: Appendix 3 Included systematic reviews. Click here for file

Additional file 4: Table S2 Summary of included systematic reviews [13-39]. Click here for file
