# Supplementary material for: Gaps in the evidence for prevention and treatment of maternal anaemia: a review of systematic reviews
Source: BMC Pregnancy Childbirth. 2012 Jun 24;12:56. doi: 10.1186/1471-2393-12-56 (PMC3475131; doi:10.1186/1471-2393-12-56)
Supplement: Additional file 1: Appendix 1 — Search narrative and strategies. [file 1471-2393-12-56-S1.doc]

**Appendix 1: Search narrative and strategies**

**SEARCH NARRATIVE**

Searches for systematic reviews of randomised controlled trials covering the prevention and treatment of anaemia in maternity were performed to 11.05.11 in the following databases:

Cochrane Database of Systematic Reviews (*The Cochrane Library* Issue 5, 2011)

DARE (*The Cochrane Library* Issue 5, 2011)

MEDLINE (1950 onwards)

EMBASE (1980 onwards)

CINAHL (1982 onwards)

British Nursing Index (1994 onwards)

UKBTS SRI Transfusion Evidence Library

MIDIRS: Maternity and Infant Care

The searches retrieved 5,177 references, which were de-duplicated and sifted down for relevance by the SRI Information Specialist to 1,378 references before sending to the lead author, Jacqui Parker.

**SEARCH STRATEGIES**

**The Cochrane Library**

1. Pregnancy explode all trees (MeSH)

2. Pregnancy Complications explode all trees (MeSH)

3. Postpartum Period explode all trees (MeSH)

4. Perinatal Care single term (MeSH)

5. Obstetrical Nursing single term (MeSH)

6. Anesthesia, Obstetrical single term (MeSH)

7. Obstetrics single term (MeSH)

8. Obstetric Surgical Procedures explode all trees (MeSH)

9. Maternal Health Services explode all trees (MeSH)

10. Maternal-Child Nursing explode all trees (MeSH)

11. Midwifery single term (MeSH)

12. Hospitals, Maternity single term (MeSH)

13. Maternal Mortality single term (MeSH)

14. Maternal Welfare single term (MeSH)

15. matern* or pregnan* or conception* or preconception* or gestat* or birth* or childbirth* or labor or laboring or labour or labouring or vaginal delivery or prenatal* or antenatal* or prenatal* or perinatal* or postnatal* or intranatal* or natal* or postpart* or prepartum or antepartum or partum* or caesar* or cesar* or obstetric* or preeclamp* or (pre next eclamp*) or eclamp* or intrapart* or placenta* or HELPP syndrome

16. #1 OR #2 OR #3 OR #4 OR #5 OR #6 OR #7 OR #8 OR #9 OR #10 OR #11 OR #12 OR #13 OR #14 OR #15

17. Anemia explode all trees (MeSH)

18. Postpartum Hemorrhage explode all trees (MeSH)

19. Hemorrhage single term (MeSH)

20. anemi* or anaemi* or iron deficien* or iron count or iron level* or low hemoglobin or low haemoglobin or low blood count* or low red cell count* or low red blood cell count* or hemorrhag* or haemorrhag* or bleed* or (blood NEAR loss) or bloodloss*

21. #17 OR #18 OR #19 OR #20

22. #16 and #21

23. ((anemi* or anaemi* or iron or low hemoglobin or low haemoglobin or low blood count or transfus* or red blood cell* or red cell* or RBC* or erythrocyte* or allogeneic blood or allogenic blood or haemorrhag* or hemorrhag* or bleed* or (blood and los*) or bloodloss*) AND (matern* or pregnan* or preconception* or conception* or gestat* or birth* or childbirth* or delivery or labor or laboring or labour* or prenatal* or antenatal* or prenatal* or perinatal* or postnatal* or intranatal* or postpart* or peripartum or antepartum or partum* caesar* or cesar* or obstetric* or preeclamp* or pre next eclamp* or eclamp* or intrapart* or placenta* or HELPP syndrome)):ti

24. #22 OR #23

**MEDLINE & MIDIRS (Ovid)**

1. exp Pregnancy/

2. exp Pregnancy Complications/

3. exp Postpartum Period/

4. Perinatal Care/

5. Obstetrical Nursing/

6. Anesthesia, Obstetrical/

7. Obstetrics/

8. exp Obstetric Surgical Procedures/

9. exp Maternal Health Services/

10. exp Maternal-Child Nursing/

11. Midwifery/

12. Hospitals, Maternity/

13. Maternal Mortality/

14. Maternal Welfare/

15. (matern* or pregnan* or conception* or preconception* or gestat* or birth* or childbirth* or labor or laboring or labour or labouring or vaginal delivery or prenatal* or antenatal* or prenatal* or perinatal* or postnatal* or intranatal* or natal* or postpart* or peripartum or antepartum or partum* or caesar* or cesar* or obstetric* or preeclamp* or (pre adj eclamp*) or eclamp* or intrapart* or placenta* or HELPP syndrome).tw.

16. or/1-15

17. exp Anemia/

18. Hemorrhage/

19. exp Postpartum Hemorrhage/

20. (anemi* or anaemi* or (iron adj1 deficien*) or iron count or iron level* or low hemoglobin or low haemoglobin or low blood count* or low red cell count* or low red blood cell count* or hemorrhag* or haemorrhag* or bleed* or (blood adj2 loss) or bloodloss*).tw.

21. or/17-20

22. 16 and 21

23. ((anemi* or anaemi* or iron or low hemoglobin or low haemoglobin or low blood count or transfus* or red blood cell* or red cell* or RBC* or erythrocyte* or allogeneic blood or allogenic blood or haemorrhag* or hemorrhag* or bleed* or (blood and los*) or bloodloss*) AND (matern* or pregnan* or conception* or preconception* or gestat* or birth* or childbirth* or delivery or labor or laboring or labour* or prenatal* or antenatal* or prenatal* or perinatal* or postnatal* or intranatal* or postpart* or post partum* or caesar* or cesar* or obstetric* or preeclamp* or pre next eclamp* or eclamp* or intrapart* or placenta* or HELPP syndrome)).ti.

24. 22 or 23

**EMBASE (Ovid)**

1. exp Pregnancy/

2. exp Childbirth/

3. exp Pregnancy Disorder/

4. exp Puerperium/

5. exp Obstetric Care/

6. Obstetrical Nursing/

7. exp Obstetrics/

8. Maternal Welfare/

9. Maternal Mortality/

10. (matern* or pregnan* or conception* or preconception* or gestat* or birth* or childbirth* or labor or laboring or labour or labouring or prenatal* or antenatal* or prenatal* or perinatal* or postnatal* or intranatal* or postpart* or post partum* or caesar* or cesar* or obstetric* or preeclamp* or pre next eclamp* or eclamp* or intrapart* or placenta* HELPP syndrome).tw.

11. or/1-10

12. exp Anemia/

13. Bleeding/ or exp Obstetric Hemorrhage/

14. (anemi* or anaemi* or (iron adj1 deficien*) or iron count or iron level* or low hemoglobin or low haemoglobin or low blood count* or low red cell count* or low red blood cell count* or hemorrhag* or haemorrhag* or bleed* or (blood adj2 loss) or bloodloss*).tw.

15. or/12-14

16. 11 and 15

17. ((anemi* or anaemi* or iron or low hemoglobin or low haemoglobin or low blood count or transfus* or red blood cell* or red cell* or RBC* or erythrocyte* or allogeneic blood or allogenic blood or haemorrhag* or hemorrhag* or bleed* or (blood and los*) or bloodloss*) AND (matern* or pregnan* or preconception* or conception* or gestat* or birth* or childbirth* or delivery or labor or laboring or labour* or prenatal* or antenatal* or prenatal* or perinatal* or postnatal* or intranatal* or postpart* or post partum* or caesar* or cesar* or obstetric* or preeclamp* or pre next eclamp* or eclamp* or intrapart* or placenta* or HELPP syndrome)).ti.

18. 16 or 17

**CINAHL & BNI (NHS Evidence)**

1. exp Pregnancy/

2. exp Pregnancy Complications/

3. exp Postnatal Period/

4. exp Obstetric Care/

5. Obstetric Emergencies/

6. exp Surgery, Obstetrical/

7. Anesthesia, Obstetrical/

8. Obstetrics/

9. exp Maternal Health Services/

10. exp Maternal-Child Nursing/

11. exp Midwifery/

12. Maternal Mortality/

13. Maternal Welfare/

14. (matern* OR pregnan* OR conception* OR preconception* or gestat* or birth* OR childbirth* OR labor OR laboring OR labour OR labouring OR "vaginal delivery" OR prenatal* OR antenatal* OR prenatal* OR perinatal* OR postnatal* OR intranatal* OR natal* OR postpart* OR peripartum OR antepartum OR partum* OR caesar* OR cesar* OR obstetric* OR preeclamp* OR "pre eclamp*" OR eclamp* OR intrapart* OR placenta* OR HELPP).ti,ab

15. or/1-14

16. exp Anemia/

17. Hemorrhage/

18. Postpartum Hemorrhage/

19. (anemi* OR anaemi* OR (iron adj1 deficien*) OR "iron store*" OR "iron count" OR "iron level*" OR "low hemoglobin" OR "low haemoglobin" OR "low blood count*" OR "low red cell count*" OR "low red blood cell count*" OR hemorrhag* OR haemorrhag* OR bleed* OR (blood adj2 loss) OR bloodloss*).ti,ab

20. or/16-19

21. 15 and 20

22. ((anemi* OR anaemi* OR iron OR "low hemoglobin" OR "low haemoglobin" OR "low blood count" OR transfus* OR "red blood cell*" OR "red cell*" OR RBC* OR erythrocyte* OR "allogeneic blood" OR "allogenic blood" OR haemorrhag* OR hemorrhag* OR bleed* OR "blood loss" OR bloodloss*) AND (matern* OR pregnan* OR preconception* or conception* or gestat* or birth* OR childbirth* OR delivery OR labor OR laboring OR labour* OR prenatal* OR antenatal* OR prenatal* OR perinatal* OR postnatal* OR intranatal* OR postpart* OR partum* OR caesar* OR cesar* OR obstetric* OR preeclamp* OR "pre eclamp*" OR eclamp* OR intrapart* OR placenta* OR HELPP)).ti

23. 21 or 22
