# Supplementary material for: Gaps in the evidence for prevention and treatment of maternal anaemia: a review of systematic reviews
Source: BMC Pregnancy Childbirth. 2012 Jun 24;12:56. doi: 10.1186/1471-2393-12-56 (PMC3475131; doi:10.1186/1471-2393-12-56)
Supplement: Additional file 3: Appendix 3 — Included systematic reviews. [file 1471-2393-12-56-S3.doc]

**Appendix 3: Included systematic reviews**

Fishman S, Christian P, West K. **The role of vitamins in the prevention and control anaemia**. *Public Health Nutr.* 2000;**3**(2):125-50.

Villar J, Merialdi M, Gulmezoglu, M, Abalos E, Carroli G, Kulier R, et al. **Nutritional interventions during pregnancy for the prevention or treatment of maternal morbidity and preterm delivery: An overview of randomized controlled trials**. *J Nutr*. 2003;**133**(5 Suppl 1):S1606-25.

Haider B, Bhutta Z. **Multiple-micronutrient supplementation for women during pregnancy**. *Cochrane Database of Systematic Reviews* 2006 (4): CD004905.

Haider B, Yakoob M, Bhutta Z. **Effect of multiple micronutrient supplementation during pregnancy on maternal and birth outcomes**. *BMC Public Health* 2011;**11**(Suppl 3):S19.

Faisel H, Pittrof R. **Vitamin A and causes of maternal mortality: Association and biological plausibility**. *Public Health Nutr.* 2000;**3**(3):321-7.

Van Den Broek, Kulier R, Gulmezoglu M, Villar J. **Vitamin A supplementation during pregnancy**. *Cochrane Database of Systematic Reviews* 2002 (4): CD001996.

Rumbold A, Crowther C. **Vitamin E supplementation in pregnancy**. *Cochrane Database of Systematic Reviews* 2005 (2): CD004069.

Rumbold A, Crowther C. **Vitamin C supplementation in pregnancy**. *Cochrane Database of Systematic Reviews* 2005 (2): CD004072.

Gulmezoglu M, De Onis M, Villar J. **Effectiveness of interventions to prevent or treat impaired fetal growth**. *Obstet Gynecol Surv.* 1997;**52**(2):139-49.

Kulier R, De Onis M, Gulmezoglu M, Villar J. **Nutritional interventions for the prevention of maternal morbidity**. *Int J Gynaecol Obstet.* 1998;**63**(3):231-46.

De Onis M, Villar J, Gulmezoglu M. **Nutritional interventions to prevent intrauterine growth retardation: evidence from randomized controlled trials**. *Eur J Clin Nutr.* 1998;**52**(Suppl1):S83-93.

Milman N, Bergholt T, Byg K, Eriksen L, Graudal N. **Iron status and iron balance during pregnancy. A critical reappraisal of iron supplementation**. *Acta Obstet et Gynecol Scand.* 1999;**78**(9): 749-57.

Rasmussen K. **Is there a causal relationship between iron deficiency or iron-deficiency anemia and weight at birth, length of gestation and perinatal mortality?** *J Nutr.* 2001;**131** (2 suppl 2):S590-603.

Sloan N, Jordan E, Winikoff B. **Effects of iron supplementation on maternal hematologic status in pregnancy**. *Am J Public Health* 2002;**92**(2):288-93.

Agency for Healthcare Research and Quality. **Screening for iron deficiency anemia in childhood and pregnancy** update of the 1996 U.S. *Preventive Task Force review.* 2006;50.

Reveiz L, Gyte G, Cuervo L. **Treatments for iron-deficiency anaemia in pregnancy**. *Cochrane Database of Systematic Reviews* 2007 (2): CD003094

Peña-Rosas J, Viteri F. **Effects and safety of preventive oral iron or iron+folic acid supplementation for women during pregnancy**. *Cochrane Database of Systematic Reviews* 2009 (4): CD004736.

Macedo A, Cardoso S. **Routine iron supplementation in pregnancy**. *Acta Med Port* 2010;**23**:785-92.

Yakoob M, Bhutta, Z. **Effect of routine iron supplementation with or without folic acid on anemia during pregnancy**. *BMC Public Health* 2011;**11**(Suppl 3):S21.

Scholl T, Hediger M, Belsky D. **Prenatal care and maternal health during adolescent pregnancy: a review and meta-analysis**. *J Adolesc Health* 1994;**15**(6):444-56.

Carroli G, Villar J, Piaggio G, Khan-Neelofur D, Gulmezoglu M, Mugford M, et al. **WHO systematic review of randomised controlled trials of routine antenatal care**. *Lancet* 2001;**357**(9268):1565-70.

Villar J, Carroli G, Khan-Neelofur D, Piaggio G, Gülmezoglu M. **Patterns of routine antenatal care for low-risk pregnancy**. *Cochrane Database of Systematic Reviews* 2001 (4): CD000934.

Carroli G, Rooney C, Villar J. **How effective is antenatal care in preventing maternal mortality and serious morbidity? An overview of the evidence**. *Paediatr Perinat Epidemiol.* 2001;**15** (suppl 1):1-42.

Dodd J, Crowther C. **Specialised antenatal clinics for women with a multiple pregnancy for improving maternal and infant outcomes**. *Cochrane Database of Systematic Reviews* 2007 (2): CD005300.

Mathews F. **Antioxidant nutrients in pregnancy: a systematic review of the literature**. *Nutrition Research Reviews* 1996;**9**:175-95.

Hodnett E, Downe S, Walsh D, Weston J. **Alternative versus conventional institutional settings for birth**. *Cochrane Database of Systematic Reviews* 2010 (9): CD000012.

Woolley R. **Benefits and risks of episiotomy: A review of the english-language literature since 1980**. *Obstet Gynecol Surv.* 1995;**50**(11):821-35.

Viswanathan M, Hartmann K, Palmieri R, Lux L, Swinson T, Lohr K, et al. **The use of episiotomy in obstetrical care: a systematic review**. *Agency for Healthcare Research and Quality* 2005;102.

Carroli G, Mignini L. **Episiotomy for vaginal birth**. *Cochrane Database of Systematic Reviews* 2009 (1): CD000081.

Guise J, Denman M, Emeis C, Marshall N, Walker M, Fu R, et al. **Vaginal birth after caesarian: new insights on maternal and neonatal outcomes**. *Obstet Gynecol.* 2010;**115**(6):1267-78.

Gupta J, Hofmeyr G, Smyth R. **Position in the second stage of labour for women without epidural anaesthesia**. *Cochrane Database of Systematic Reviews* 2009 (4): CD002006.

Altman M, Lydon-Rochelle M. **Prolonged second stage of labor and risk of adverse maternal and perinatal outcomes: a systematic review**. *Birth* 2006;**33**(4):315-22.

Althabe F, Bergel E, Buekens P, Sosa C, Belizan J. **Controlled cord traction in the third stage of labor Systematic review**. *Int J Gynecol Obstet.* 2006;**94**(suppl 2):S126-7.

Peña-Martí G, Comunián-Carrasco G. **Fundal pressure versus controlled cord traction as part of the active management of the third stage of labour**. *Cochrane Database of Systematic Reviews* 2007 (4): CD005462.

Prendiville W, Elbourne D, Chalmers I. **The effects of routine oxytocic administration in the management of the third stage of labour: an overview of the evidence from controlled trials**. *Br Journal Obstet Gynaecol.* 1988;**95**(1):3-16.

Prendiville W. **The prevention of post partum haemorrhage: optimising routine management of the third stage of labour**. *Eur J Obstet Gynecol Reprod Biol.* 1996;**69**(1):19-24.

Cotter A, Ness A, Tolosa J. **Prophylactic oxytocin for the third stage of labour**. *Cochrane Database of Systematic Reviews* 2001 (4): CD001808.

Joy S, Sanchez-Ramos L, Kaunitz A. **Misoprostol use during the third stage of labor**. *Int J Gynecol Obstet.* 2003;**82**(2):143-52.

McDonald S, Abbott J, Higgins S. **Prophylactic ergometrine-oxytocin versus oxytocin for the third stage of labour**. *Cochrane Database of Systematic Reviews* 2004 (1): CD000201.

Van Liabsuetrakul T, Choobun T, Peeyananjarassri K, Islam M. **Prophylactic use of ergot alkaloids in the third stage of labour**. *Cochrane Database of Systematic Reviews* 2007 (2): CD005456.

Begley C, Gyte G, Murphy D, Devane D, McDonald S, McGuire W. **Active versus expectant management for women in the third stage of labour**. *Cochrane Database of Systematic Reviews* 2010 (7): CD007412.

Abdel-Aleem H, Abdel-Aleem M, Shaaban O. **Tocolysis for management of retained placenta**. *Cochrane Database of Syatematic Reviews* 2001 (1): CD007708.

Van Rheenen P, Brabin B. **A practical approach to timing cord clamping in resource poor settings**. *British Medical Journal* 2006;**333**(7575):954-8.

McDonald S, Middleton P. **Effect of timing of umbilical cord clamping of term infants on maternal and neonatal outcomes**. *Cochrane Database of Systematic Reviews* 2008 (2): CD004074.

Mathew J. **Timing of umbilical cord clamping in term and preterm deliveries and infant and maternal outcomes: A systematic review of randomized controlled trials**. *Indian Pediatrics* 2011;**48**(2):123-9.

Carroli G, Bergel E. **Umbilical vein injection for management of retained placenta**. *Cochrane Database of Systematic Reviews* 2001 (4): CD001337.

Soltani H, Dickinson F, Leung T. **The effect of placental cord drainage in the third stage of labour on feto-maternal transfusion: a systematic review**. *Evidence Based Midwifery* 2005;**3**(2):64-9.

Villar J, Gulmezoglu M, Hofmeyr G, Forna F. **Systematic review of randomized controlled trials of misoprostol to prevent postpartum hemorrhage**. *Obstet Gynecol.* 2002;**100**(6): 1301-12.

Kuala Lumpur; Malaysian Health Technology Assessment Unit (Mhtau). **Misoprostol in pregnancy** 2004.

Hofmeyr G, Walraven G, Gulmezoglu M, Maholwana B, Alfirevic Z, Villar J. **Misoprostol to treat postpartum haemorrhage: a systematic review**. *Br J Obstet Gynaecol*. 2005;**112**(5):547-53.

Langenbach C. **Misoprostol in preventing postpartum hemorrhage: a meta-analysis**. *Int J Gynaecol Obstet* 2006;**92**(1):10-8.

Gülmezoglu M, Forna F, Villar J, Hofmeyr G. **Prostaglandins for preventing postpartum haemorrhage** *Cochrane Database of Systematic Reviews* 2007 (3): CD000494.

Wei S, Fraser W. **Review: misoprostol and intramuscular prostaglandins do not prevent postpartum haemorrhage more than injectable uterotonics**. *Evidence-Based Medicine* 2008;**13**(3):82-3.

Hofmeyr G, Gulmezoglu M, Novikova N, Linder V, Ferreira S, Piaggio G. **Misoprostol to prevent and treat postpartum haemorrhage: a systematic review and meta-analysis of maternal deaths and dose-related effects**. *Bulletin of the World Health Organization* 2009;**87**(9):666-77.

Su L, Chong Y, Samuel M. **Oxytocin agonists for preventing postpartum haemorrhage**. *Cochrane Database of Systematic Reviews* 2007 (3): CD005457.

Peters N, Duvekot J. **Carbetocin for the prevention of postpartum hemorrhage: a systematic review**. *Obstet Gynecol Surv.* 2009;**64**(2):129-35.

Mousa H, Alfirevic Z. **Treatment for primary postpartum haemorrhage**. *Cochrane Database of Systematic Reviews* 2007 (1): CD003249.

Sloan N, Durocher J, Aldrich T, Blum J, Winikoff B. **What measured blood loss tells us about postpartum bleeding: a systematic review**. *Br J Obstet Gynaecol.* 2010;**117**(7): 788-800.

Hofmeyr G, Abdel-Aleem H, Abdel-Aleem M. **Uterine massage for preventing postpartum haemorrhage**. *Cochrane Database of Systematic Reviews* 2008 (3): CD006431.

Miller S, Ojengbede A, Turan J, Ojengbede O, Butrick E, Hensleigh P. **Anti-shock garments for obstetric hemorrhage**. *Current Women's Health Reviews* 2007;**3**(1):3-11.

Ferrer P, Roberts I, Sydenham E, Blackhall K, Shakur H. **Anti-fibrinolytic agents in post partum haemorrhage: a systematic review**. *BMC Pregnancy and Childbirth* 2009;**9**: 29.

Sloan N, Durocher J, Aldrich T, Blum J, Winikoff B. **What measured blood loss tells us about postpartum bleeding: a systematic review**. *Br J Obstet Gynaecol.* 2010;**117**(7):788-800.

Novikova N, Hofmeyr G. **Tranexamic acid for preventing postpartum haemorrhage**. *Cochrane Database of Systematic Reviews* 2009 (4): CD007872

Franchini M, Franchi M, Bergamini V, Salvagno L, Montagnana M, Lippi G. **A critical review on the use of recombinant factor VIIa in life-threatening obstetric postpartum hemorrhage**. *Semin Thromb Hemost.* 2008;**34**(1):104-12.

Franchini M, Franchi M, Bergamini V, Montagnana M, Salvagno G, Targher G et al. **The use of recombinant activated FVII in postpartum hemorrhage**. *Clin Obstet Gynecol.* 2010;**53**(1):219-27.

Rossi A, Lee R, Chmait R. **Emergency postpartum hysterectomy for uncontrolled postpartum bleeding: a systematic review**. *Obstet Gynecol.* 2010;**115**(3):637-44.

Delotte J, Novellas S, Ko, C, Bongain A, Chevallier P. **Obstetrical prognosis and pregnancy outcome following pelvic arterial embolisation for post-partum hemorrhage**. *Eur J Obstet Gynecol Reprod Biol* 2009;**145**(2):129-32 .

Tessier V, Pierre F. **Risk factors of postpartum hemorrhage during labor and clinical and pharmacological prevention**. *J Gynecol Obstet Biol Reprod.* 2004;**33**(suppl 8):4S29-56.

Doumouchtsis S, Papageorghiou A, Arulkumaran S. **Systematic review of conservative management of postpartum hemorrhage: what to do when medical treatment fails**. *Obstet Gynecol Surv.* 2007;**62**(8): 540-7.

Chelmow D. **Postpartum haemorrhage: prevention**. *Clinical Evidence* 2011.

Alderdice F, McKenna D, Dornan J. **Techniques and materials for skin closure in caesarean section**. *Cochrane Database of Systematic Reviews* 2003 (2): CD003577.

Anderson E, Gates S. **Techniques and materials for closure of the abdominal wall in caesarean section**. *Cochrane Database of Systematic Reviews* 2004 (4): CD004663.

Jacobs-Jokhan D, Hofmeyr G. **Extra-abdominal repair of the uterine incision at caesarian section**. *Cochrane Database of Systematic Reviews* 2004 (4): CD000085.

Berghella V, Baxter J, Chauhan S. **Evidence-based surgery for cesarean delivery**. *Am J Obstet Gynecol.* 2005;**193**(5):1607-17.

Gates S, Anderson E. **Wound drainage for caesarean section**. *Cochrane Database of Systematic Reviews* 2005 (1): CD004549.

Dodd J, Anderson E, Gates S. **Surgical techniques for uterine incision and uterine closure at the time of caesarean section**. *Cochrane Database of Systematic Reviews* 2008 (3): CD004732.

Hellums E, Lin M, Ramsey P. **Prophylactic subcutaneous drainage for prevention of wound complications after cesarean delivery: a metaanalysis**. *Am J Obstet Gynecol.* 2007;**197**(3): 229-35.

Anorlu R, Maholwana B, Hofmeyr G. **Methods of delivering the placenta at caesarean section**. *Cochrane Database of Systematic Reviews* 2008 (3): CD004737.

Hofmeyr G, Mathai M, Shah A, Novikova N. **Techniques for caesarean section**. *Cochrane Database of Systematic Reviews* 2008 (1): CD004662.

Dodd J, Reid K. **Tocolysis for assisting delivery at caesarean section**. *Cochrane Database of Systematic Reviews* 2006 (4): CD004944.

Hofmeyr G, Novikova N, Mathai M, Shah A. **Techniques for cesarean section**. *Am J Obstet Gynecol.* 2009;**201**(5):431-44.

Mathai M, Hofmeyr G. **Abdominal surgical incisions for caesarean section**. *Cochrane Database of Systematic Reviews* 2007 (1): CD004453.

Walsh C, Walsh S. **Extraabdominal vs intraabdominal uterine repair at cesarean delivery: a metaanalysis**. *Am J Obstet Gynecol.* 2009;**200**(6):625.e1-8.

Visco A, Viswanathan M, Lohr K, Wechter M, Gartlehner G, Wu J, et al. **Cesarean delivery on maternal request: maternal and neonatal outcomes**. *Obstet Gynecol.* 2006;**108**(6):1517-29.

Neilson J. **Interventions for suspected placenta praevia**. *Cochrane Database of Systematic Reviews* 2003 (2): CD001998.

Oyelese Y, Smulian J. **Placenta previa, placenta accreta, and vasa previa**. *Obstet Gynecol.* 2006;**107**(4):927-41.

Neilson J. **Interventions for treating placental abruption**. *Cochrane Database of Systematic Reviews* 2003 (1): CD003247

Dunn C, Goa K. **Tranexamic acid: a review of it’s use in surgery and other indications**. *Drugs* 1999;**57**(6):1005-32.

Peitsidis P, Kadir R. **Antifibrinolytic therapy with tranexamic acid in pregnancy and postpartum**. *Expert Opinion on Pharmacotherapy* 2011;**12**(4):503-16.

Allam J, Cox M, Yentis S. **Cell salvage in obstetrics**. *Int J Obstet Anes.* 2008;**17**(1):37-45.

Dodd J, Dare M, Middleton P. **Treatment for women with postpartum iron deficiency anaemia**. *Cochrane Database of Systematic Reviews* 2004 (4): CD004222.

Kotto-Kome A, Calhoun D, Montenegro R, Sosa R, Maldonado L, Christensen R. **Effect of administering recombinant erythropoietin to women with postpartum anemia: a meta-analysis**. *J Perinatol.* 2004;**24**(1):11-5.
