# Supplementary material for: Gaps in the evidence for prevention and treatment of maternal anaemia: a review of systematic reviews
Source: BMC Pregnancy Childbirth. 2012 Jun 24;12:56. doi: 10.1186/1471-2393-12-56 (PMC3475131; doi:10.1186/1471-2393-12-56)
Supplement: Additional file 4: Table S2 — Summary of included systematic reviews [13-39]. [file 1471-2393-12-56-S4.doc]

**Table 2: Summary of included systematic reviews**

**The prevention of anaemia in the antenatal period**

Nutritional intervention

| **Author**  **Publication year**  ***(Search date)*** | **Stated aim of review** | **No. & type of studies included** | **No. & type of studies included with outcomes relevant to anaemia** | **Outcomes measured:**  **Clinical** | **Outcomes measured:**  **Laboratory** |
| --- | --- | --- | --- | --- | --- |
| Fishman, S *  [16]  (1967-2000) | The role of vitamin supplementation in prevention and control of anaemia | 62 RCTs | 26 RCTs | None | Change in mean haemoglobin, mean haematocrit, serum iron and ferritin |
| Villar, J  [17]  *(Up to July 2002)* | Nutritional interventions for prevention of maternal morbidity and pre-term delivery | 13 SR (of RCTs)  1 RCT | 29 RCTs | Hypertension, haemorrhage,caesarean section rates | Haemoglobin |
| Haider, B ↑  [18]  *(Up to 2005)* | Benefits and risks to mother and infant of multiple micronutrient supplementation in pregnancy | 9 RCTs | 6 RCTs | Birth weight, small for gestational age, preterm birth, perinatal mortality | Maternal anaemia unspecified |
| Haider, B ↑  [19]  *(Up to Dec 2009)* | Impact of multiple micronutrient supplementation in pregnancy on maternal and birth outcomes | 17 RCTs | 4 RCTs | Small for gestational age, neonatal mortality | Maternal anaemia unspecified |
| Faisel, H  [20]  *(1981-1999)* | Association between vitamin A supplementation and maternal mortality | Not stated | 2 RCTs | None | Maternal anaemia unspecified  Iron, transferrin |
| Van den Broek ↑  [21]  *(Up to April 2002)* | Effectiveness of vitamin A supplementation | 5 RCTs | 5 RCTs | Outcomes sought but not found | Haemoglobin |
| Rumbold, A ↑  [22]  *(Up to June 2004)* | To assess the effects of vitamin E supplementation | 4 RCTs | 0 | None | None |
| Rumbold, A ↑  [23]  *(Up to June 2004)* | To assess the effects of vitamin C supplementation | 5 RCTs | 0 | None | None |
| Gulmezoglu, M [24]  *(Up to 1996)* | Evaluate 36 antenatal interventions on fetal growth | 126 RCTs | Unclear | Term low birth weight | Haemoglobin ferritin |
| Kullier, R  [25]  *(Up to 1998)* | Nutritional interventions for prevention of maternal morbidity | 31 RCTs | 9 RCTs | Hypertension, blood transfusion, caesarean section rates | Haemoglobin |
| De Onis, M  [26]  *(Up to March 1997)* | Nutritional interventions for prevention of intrauterine growth retardation | 12 SR (of RCTS)  1 RCT | 2 SR (=7 RCTs) | Term low birth weight | Haemoglobin, serum ferritin |
| Milman, N  [27]  *(?)* | Summarize knowledge of iron supplementation in pregnancy | 10 RCTs | 10 RCTs | None | Haemoglobin, maternal & infant serum ferritin |
| Rasmussen, K *  [28]  *(1966-1999)* | Is IDA, or anaemia from any cause are causally related to low birth weight, preterm birth, or mortality | 23 RCTs | 23 RCTs | Birthweight, gestational age, perinatal mortality | None |
| Sloan, N  [29]  *(1966-1998)* | Effect of iron supplementation on maternal haemoglobin levels | 23 RCTs | 23 RCTs | Gastric side effects | Haemoglobin |
| AHRQ *  [30]  *(Up to 2005)* | Iron supplementation outcomes, adverse events | Not stated | Unclear | Birthweight, length of gestation, caesarean section rates, blood transfusion | Haemoglobin |
| Reveiz, L * ↑  [31]  *(Up to Jan 2007)* | Effects of different iron preparations and regimes on maternal anaemia | 17 RCTs | 17 RCTs | Adverse effects other outcomes sought but Not found | Haemoglobin, ferritin, |
| Pena-Rosas, J ↑  [32]  *(Up to March 2009)* | Effectiveness and safety of daily and intermittent iron, iron and folic acid supplementation | 49 RCTs | 49 RCTs | Low birthweight, birth length, maternal wellbeing others sought not found | Haemoglobin, infant ferritin |
| Macedo, A  [33]  *(Up to Aug 2008)* | Clinical effects of routine iron supplementation on healthy uncomplicated pregnancies | 3 practice guidelines  2 MA  3 SR  7 RCTs | 3 practice guidelines  2 MA  3 SR  7 RCTs | Preterm labor, duration of pregnancy, LSCS rate, birth weight, perinatal mortality, Apgar at birth | Haemoglobin, iron and ferritin |
| Yakoob, M ↑  [34]  *(Up to June 2010)* | Assess the efficacy of iron and folic acid supplementation in preventing anaemia in pregnancy | 31 RCTs | 31 RCTs | None | Haemoglobin, indicators of iron deficiency |

Organisation of antenatal care

| **Author**  **Publication year**  ***(Search date)*** | **Stated aim of review** | **No. & type of studies included** | **No. & type of studies included with outcomes relevant to anaemia** | **Outcomes measured:**  **Clinical** | **Outcomes measured:**  **Laboratory** |
| --- | --- | --- | --- | --- | --- |
| Scholl T  [13]  *(Up to 1993)* | Effectiveness of specialist antenatal care for adolescent mothers | Not stated | 7 RCTs | None | Haemoglobin |
| Carroli G, Villar J ↑  [35]  *(Up to June 2000)* | Assessing the effectiveness of different models of antenatal care | 7 RCTs | 1 RCT | None | Postpartum anaemia unspecified |
| Villar J ↑  [36]  *(Up to May 2001)* | Effects of antenatal care program for low risk women | 10 RCTs | 1 RCT | None | Postpartum anaemia unspecified |
| Carroli G, Rooney C  [37]  *(Up to 2000)* | Effectiveness of antenatal care in relation to maternal mortality and serious morbidity | Not stated | Not stated | None | Haemoglobin |
| Dodd J ↑  [38]  *(Up to October 2006)* | Effectiveness of specialist clinics for multiple pregnancies | 0 | 0 | None | None |

**The treatment of anaemia in the antenatal period**

Nutritional supplementation

| **Author**  **Publication year**  ***(Search date)*** | **Stated aim of review** | **No. & type of studies included** | **No. & type of studies included with outcomes relevant to anaemia** | **Outcomes measured:**  **Clinical** | **Outcomes measured:**  **Laboratory** |
| --- | --- | --- | --- | --- | --- |
| Mathews, F  [39]  *(1984-Nov 1995)* | The role of antioxidant nutrients in pregnancy | Unclear | 1 RCT  4 Cohort | Birth weight, gestational age | None |
| Rasmussen, K *  [28]  *(1966-1999)* | Is IDA, or anaemia from any cause are causally related to low birth weight, preterm birth, or mortality | 23 RCTs | 23 RCTs | Birthweight, gestational age, perinatal mortality | None |
| Reveiz, L * ↑  [31]  *(Up to Jan 2007)* | Effects of different iron preparations and regimes on maternal anaemia | 17 RCTs | 17 RCTs | Adverse effects other outcomes sought but not found | Haemoglobin, ferritin, |

The prevention of anaemia in the postnatal period

| **Author**  **Publication year**  ***(Search date)*** | **Stated aim of review** | **No. & type of studies included** | **No. & type of studies included with outcomes relevant to anaemia** | **Outcomes measured:**  **Clinical** | **Outcomes measured:**  **Laboratory** |
| --- | --- | --- | --- | --- | --- |
| Fishman, S *  [16]  (1967-2000) | The role of vitamin supplementation in prevention and control of anaemia | 62 RCTs | 26 RCTs | None | Change in mean haemoglobin, mean haematocrit, serum iron and ferritin |

The treatment of anaemia in the postnatal period

| **Author**  **Publication year**  ***(Search date)*** | **Stated aim of review** | **No. & type of studies included** | **No. & type of studies included with outcomes relevant to anaemia** | **Outcomes measured:**  **Clinical** | **Outcomes measured:**  **Laboratory** |
| --- | --- | --- | --- | --- | --- |
| Dodd, J ↑  [14]  *(Up to May 2004)* | Assess clinical effects of treatments for postpartum anaemia | 6 RCTs | 6 RCTs | Postpartum depression, lactation, blood transfusion | Haemoglobin, haematocrit |
| Kotto-Kome, A  [15]  *(Dec 2002)* | Effect of recombinant erythropoietin administration in the postpartum period | 8 RCTs  1 CS | 8 RCTs  1 CS | None | Haemoglobin |
| AHRQ *  [30]  *(Up to 2005)* | Iron supplementation outcomes, adverse events | Not stated | Unclear | Birthweight, length of gestation, caesarean section rates, blood transfusion | Haemoglobin |

* Systematic review included in more than one table

↑ Systematic review categorised as high quality
